# Identifying Key Predictors of Cognitive Dysfunction in Older People Using Supervised Machine Learning Techniques: Observational Study

**DOI:** 10.2196/20995

**Published:** 2020-09-16

**Authors:** Debbie Rankin, Michaela Black, Bronac Flanagan, Catherine F Hughes, Adrian Moore, Leane Hoey, Jonathan Wallace, Chris Gill, Paul Carlin, Anne M Molloy, Conal Cunningham, Helene McNulty

**Affiliations:** 1 School of Computing, Engineering and Intelligent Systems Ulster University Derry~Londonderry United Kingdom; 2 School of Biomedical Sciences Nutrition Innovation Centre for Food and Health Ulster University Coleraine United Kingdom; 3 School of Geography and Environmental Sciences Ulster University Coleraine United Kingdom; 4 School of Computing Ulster University Jordanstown United Kingdom; 5 School of Health, Wellbeing and Social Care The Open University Belfast United Kingdom; 6 School of Medicine Trinity College Dublin Dublin Ireland; 7 Mercers Institute for Research on Ageing St James's Hospital Dublin Ireland

**Keywords:** classification, supervised machine learning, cognition, diet, aging, geriatric assessment

## Abstract

**Background:**

Machine learning techniques, specifically classification algorithms, may be effective to help understand key health, nutritional, and environmental factors associated with cognitive function in aging populations.

**Objective:**

This study aims to use classification techniques to identify the key patient predictors that are considered most important in the classification of poorer cognitive performance, which is an early risk factor for dementia.

**Methods:**

Data were used from the Trinity-Ulster and Department of Agriculture study, which included detailed information on sociodemographic, clinical, biochemical, nutritional, and lifestyle factors in 5186 older adults recruited from the Republic of Ireland and Northern Ireland, a proportion of whom (987/5186, 19.03%) were followed up 5-7 years later for reassessment. Cognitive function at both time points was assessed using a battery of tests, including the Repeatable Battery for the Assessment of Neuropsychological Status (RBANS), with a score <70 classed as poorer cognitive performance. This study trained 3 classifiers—decision trees, Naïve Bayes, and random forests—to classify the RBANS score and to identify key health, nutritional, and environmental predictors of cognitive performance and cognitive decline over the follow-up period. It assessed their performance, taking note of the variables that were deemed important for the optimized classifiers for their computational diagnostics.

**Results:**

In the classification of a *low* RBANS score (<70), our models performed well (*F*_1_ score range 0.73-0.93), all highlighting the individual’s score from the Timed Up and Go (TUG) test, the age at which the participant stopped education, and whether or not the participant’s family reported memory concerns to be of key importance. The classification models performed well in classifying a greater rate of decline in the RBANS score (*F*_1_ score range 0.66-0.85), also indicating the TUG score to be of key importance, followed by blood indicators: plasma homocysteine, vitamin B6 biomarker (plasma pyridoxal-5-phosphate), and glycated hemoglobin.

**Conclusions:**

The results suggest that it may be possible for a health care professional to make an initial evaluation, with a high level of confidence, of the potential for cognitive dysfunction using only a few short, noninvasive questions, thus providing a quick, efficient, and noninvasive way to help them decide whether or not a patient requires a full cognitive evaluation. This approach has the potential benefits of making time and cost savings for health service providers and avoiding stress created through unnecessary cognitive assessments in low-risk patients.

## Introduction

Globally, populations are aging. By 2050, it is estimated that more than 2 billion people will be aged over 60 years [1]. Cognitive function generally declines with age and ranges in severity from mild cognitive impairment (MCI) to dementia. MCI can be defined as cognitive decline greater than that expected for an individual’s age and education level, but it does not interfere with activities of daily living, whereas dementia profoundly impacts normal functioning [2,3]. Dementia currently affects 50 million people worldwide, and it is estimated that this will increase to 152 million by 2050. The annual cost of dementia is estimated at US $1 trillion and is expected to more than double by 2030 [4]. Therefore, strategies that promote better brain health and well-being in older age are an urgent public health priority.

Alzheimer disease is the most common form of dementia, with other forms including vascular dementia, dementia with Lewy bodies, frontotemporal dementia, and mixed dementia. Risk factors for dementia are disease dependent but commonly include age, genetics and medical conditions including cardiovascular disease and diabetes, diet, lifestyle, and environmental factors [5]. An important recent report highlighted the complexity of dementia and the potential to prevent or delay the onset of the disease through interventions targeted at modifiable risk factors [6]. In particular, nutrition has been identified as a key area of interest, and emerging evidence links lower levels of certain vitamins with cognitive dysfunction in older adults, whereas certain dietary patterns and components appear to have protective roles in maintaining cognitive health [7].

The application of data mining within health care has become increasingly popular, driven particularly by the large amount of complex data available that test the capabilities of traditional statistical approaches [8]. In health care, as in other areas, data mining has provided a means of accessing and analyzing large volumes of data to better inform and drive change. Classification models, in particular, have been utilized extensively in the understanding of MCI. These models can help us to understand patterns in the behavior of data in terms of diagnosing MCI, specifically in the consideration of key features pertaining to a diagnosis of impairment [9,10] or predicting the progression of the impairment [11]. Furthermore, models have been developed to apply a more objective approach to the MCI diagnosis [12], not to undermine but rather to support a clinician’s analysis [13]. Na c [14] investigated the use of noninvasive, easy-to-collect variables that are commonly collected in community health care settings such as sociodemographic, health, functional, and interpersonal variables, for the prediction of cognitive impairment among community-dwelling older adults, using the Korean Longitudinal Study of Aging (KLoSA) data set [15] and a gradient boosting machine classifier.

Many studies apply machine learning approaches to the popular Open Access Series of Imaging Studies [16], Alzheimer Disease Neuroimaging Initiative (ADNI) [17], and Australian Imaging Biomarkers and Lifestyle Flagship Study of Aging (AIBL) [18] data sets consisting of neuroimaging data (eg, magnetic resonance imaging [MRI] and positron emission tomography scan data) from participants ranging from no cognitive impairment to MCI to Alzheimer disease [19]. These data sets also include a range of demographic, biomarker, clinical, and cognitive assessment data. Ding et al [20] used a Bayesian network approach for the classification of Alzheimer disease with heterogeneous features from the AIBL data set and demonstrated that machine learning could be used to select features and their appropriate combinations that are relevant for Alzheimer disease severity classification with high accuracy. Korolev et al [21] used a kernel-based classifier and the ADNI data set to develop a prognostic model for predicting MCI-to-dementia progression over a 3-year period.

The aim of our study is to compare the selection of data analytics techniques to identify determinants of cognitive health in community-dwelling older adults using existing data from the Trinity-Ulster and Department of Agriculture (TUDA) study (ClinicalTrials.gov identifier: NCT02664584). The TUDA study was designed to investigate nutritional, health, and lifestyle factors in the development of diseases related to aging, including dementia. A range of analytical models on the data were developed to determine factors that may predict poorer cognitive performance and cognitive decline over time, assessed using an in-depth neuropsychiatric test.

## Methods

### Cross-Industry Process for Data Mining Methodology

In this study, the widely used cross-industry process for data mining (CRISP-DM) research methodology was adopted [22]. CRISP-DM has 6 main steps: business understanding, data understanding, data preparation, modeling, evaluation, and deployment. In the business understanding phase, the objective of this study was to use classification techniques to identify the key patient predictors considered most important in the classification of cognitive dysfunction, which itself is a predictor of dementia. In the data understanding phase, the data quality was examined to understand data collection methods and the features contained within the TUDA data set, as described in the next section (The Data). In the data preparation phase, the TUDA data set was preprocessed to cleanse the data set and select features relevant to the modeling phase. Feature selection methods and the results of feature selection are described in the subsequent sections (*The Data* and *Feature Selection* sections in *Methods* and the *Feature Selection* section in *Results*). In the modeling phase, a number of machine learning modeling techniques were selected and applied to the prepared data and their parameters were calibrated to optimal values to increase the knowledge extracted from the data (described in the Machine Learning Techniques section in Methods and the RBANS Classification and Classifying Cognitive Decline Using the Rate of Change in the RBANS Score sections in Results). Upon building the models that produced the highest quality knowledge from the data analysis perspective, the models were thoroughly evaluated to ensure robustness and achievement of the business objectives. The knowledge gained from the models was then presented to clinical experts in a way that could be used and understood.

### The Data

The TUDA cohort provides detailed nutrition and health data, along with related lifestyle, clinical, and biochemical details, on a total of 5186 community-dwelling older adults aged 60 to 102 years, making this cohort one of the most comprehensively characterized cohorts of its kind for aging research internationally. With an overall goal to address the prevention of age-related diseases, the TUDA study is aimed at investigating nutrition and related factors in the development of common diseases of aging. TUDA study participants were recruited between 2008 and 2012 from hospital outpatient or general practice clinics in the Republic of Ireland or Northern Ireland via standardized protocols for participant sampling, assessment, and data recording and with a centralized laboratory analysis. In brief, the inclusion criteria for the TUDA study were being born on the island of Ireland, aged >60 years, and not having an existing diagnosis of dementia. Nonfasting blood samples were collected from all participants, and a wide range of parameters including routine biochemistry and hematological profiles, along with biomarkers of micronutrient status, were measured. A comprehensive health and lifestyle questionnaire was administered as part of the 90-min interview to capture medical and demographic details, along with comprehensive information on medication and vitamin supplement usage. Physiological function tests, blood pressure, bone health (dual-energy x-ray absorptiometry scans), and cognitive function tests were also performed. A subset of approximately 19.03% (987/5186) of participants were reassessed 5 to 7 years after their initial assessment to investigate the progression of risk factors and disease over time.

A summary of the characteristics of the subset of the TUDA cohort (n=2869) analyzed in this study is shown in Table 1. Preprocessing and feature selection performed on the original data set to reach this subset of data are described in the *Feature Selection* sections of the Methods and Results sections.

Cognitive function was assessed at both time points using 3 assessment tools, the Mini-Mental State Examination (MMSE), the Frontal Assessment Battery (FAB), and RBANS, and the rate of cognitive decline was calculated over the 5- to 7-year follow-up period. For the purposes of this study, the cognitive function outcome indicator is categorized based on RBANS. RBANS is an age-adjusted and sensitive neuropsychiatric battery for assessing global cognitive function [23]. This tool has also been validated to assess specific cognitive domains within the brain, including immediate and delayed memory, visual-spatial, language, and attention, which are combined to provide a total score, with lower scores generally indicative of poorer cognitive performance.

**Figure 1:**
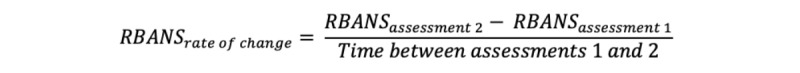
Calculating Repeatable Battery for the Assessment of Neuropsychological Status rate of change over a 5- to 7-year period between initial assessment and follow-up assessment, normalized to account for the time between each assessment.

The rate of RBANS change over the 5- to 7-year period between the initial assessment and the follow-up assessment was computed as the difference between a participant’s RBANS score at each sampling point, normalized to account for the time between each assessment, where this can differ by up to 2 years across participants (Figure 1).

**Table 1:** General characteristics of the Trinity-Ulster and Department of Agriculture study participants.

Characteristics		Males (n=1191)		Females (n=1678)	
Age (years), mean (SD)		72.1 (7.8)		72.2 (7.8)	
Education (years)^a^, mean (SD)		16.3 (3.3)		16.1 (2.8)	
**Health and lifestyle**					
	BMI (kg/m^2^), mean (SD)		28.9 (4.3)		28.7 (5.7)
	Waist-to-hip ratio, mean (SD)		0.97 (0.07)		0.88 (0.07)
	Instrumental activities of daily living, mean (SD)		25.0 (4.1)		24.9 (3.5)
	Physical self-maintenance scale score, mean (SD)		23.3 (1.6)		23.1 (1.7)
	Timed Up and Go (seconds), mean (SD)		12.9 (9.1)		13.0 (8.0)
	Living alone, n (%)		260 (21.8)		632 (37.7)
	Current smoker, n (%)		122 (10.2)		194 (11.6)
	Alcohol (units/week), mean (SD)		8.8 (14.6)		2.9 (6.7)
	Socioeconomically most deprived, n (%)		291 (24.4)		426 (25.4)
**Neuropsychiatric assessment**					
	MMSE^b^ score, mean (SD)		27.8 (1.4)		27.9 (1.4)
	RBANS^c^ score, mean (SD)		87.3 (14.5)		88.9 (15.2)
	RBANS class=“low” (target), n (%)^d^		133 (11.2)		168 (10.0)
	RBANS class=“high” (target), n (%)^d^		1058 (88.8)		1510 (90.0)
	FAB^e^ score, mean (SD)		15.7 (2.2)		15.9 (2.1)
	Depression CES-D^f^ score, mean (SD)		4.8 (6.2)		6.1 (7.7)
	Anxiety (HADS^g^ score), mean (SD)		2.6 (3.2)		3.5 (3.8)
**Clinical measures**					
	White cell count (10^9^/L), mean (SD)		7.1 (3.6)		6.9 (3.3)
	Hemoglobin (g/DL), mean (SD)		14.2 (1.5)		13.0 (1.3)
	Mean corpuscular volume (FL^h^), mean (SD)		90.7 (5.5)		90.6 (5.1)
	Platelet count (10^9^/L), mean (SD)		229 (59.0)		265 (66.9)
	Urea (mmol/L), mean (SD)		7.2 (2.9)		6.7 (2.3)
	Creatinine (μmol/L), mean (SD)		98 (31.0)		79 (22.4)
	Albumin (g/L), mean (SD)		42 (3.7)		42 (3.4)
	Gamma GT (U/L), mean (SD)		43 (47.5)		34 (36.0)
	Sodium (mmol/L), mean (SD)		140 (5.1)		139 (3.2)
	Potassium (mmol/L), mean (SD)		4.3 (0.5)		4.2 (0.4)
	Calcium (mmol/L), mean (SD)		2.3 (0.1)		2.3 (0.1)
	Phosphate (mmol/L), mean (SD)		1.0 (0.2)		1.1 (0.2)
	Alkaline phosphatase (U/L), mean (SD)		82 (34.2)		82 (25.7)
	Low-density lipoprotein (mmol/L), mean (SD)		2.23 (0.8)		2.58 (0.9)
	High-density lipoprotein (mmol/L), mean (SD)		1.23 (0.4)		1.55 (0.4)
	Triglycerides (mmol/L), mean (SD)		1.78 (1.0)		1.62 (1.0)
	C-reactive protein (mg/L), mean (SD)		6.1 (11.1)		5.5 (11.9)
	Glycated hemoglobin (%), mean (SD)		6.0 (1.0)		5.9 (0.7)
	Parathyroid hormone (pg/mL), mean (SD)		45.2 (30.8)		47.2 (31.9)
	Glomerular filtration rate (mL/min), mean (SD)		77.2 (25.3)		67.8 (22.6)
**Nutritional biomarkers**					
	Red blood cell folate (nmol/L), mean (SD)		1053 (591.1)		1100 (582.7)
	Serum vitamin B12 (pmol/L), mean (SD)		267 (191.0)		296 (277.3)
	Plasma vitamin B6 (nmol/L), mean (SD)		74.1 (53.2)		81.5 (69.7)
	Riboflavin (EGRac^i^), mean (SD)		1.35 (0.2)		1.34 (0.2)
	Total plasma homocysteine (μmol/L), mean (SD)		15.1 (5.9)		14.1 (5.1)
	Total vitamin D (nmol/L), mean (SD)		51.6 (25.9)		56.0 (30.1)

^a^Education refers to the age of stopping formal education.

^b^MMSE: Mini-Mental State Examination.

^c^RBANS: Repeatable Battery for the Assessment of Neuropsychological Assessment.

^d^RBANS score <70 is assigned class *low* and an RBANS score ≥70 is assigned class *high*.

^e^FAB: Frontal Assessment Battery.

^f^CES-D: Centre for Epidemiological Studies Depression.

^g^HADS: Hospital Anxiety and Depression Scale.

^h^FL: femtolitre.

^i^EGRac: erythrocyte glutathione reductase activation coefficient, with a higher EGRac value indicating poorer riboflavin status.

The data set initially contained 525 variables. During preprocessing, the data were cleansed to detect and correct inaccurate values, identify missing values and ensure consistent coding of these, ensure consistent coding of categorical variables, identify spelling and coding inconsistencies and correct these, transform text variables into categorical variables where possible, ensure numeric values fell within an appropriate and accurate range, check for consistency among dependent variables and correct any errors, and finally check for duplicate data and remove any redundancy. Normalization was carried out on the data table, including nonloss decomposition to decompose the large data table into smaller tables, transforming composite attributes into separate attributes, transforming multivalued attributes, repeating columns into separate tables, and recoding text attributes to categorical attributes where possible. This process reduced the number of variables to 345 within the data set. These variables were a combination of text, categorical, and numerical variables.

### Feature Selection

Dimension reduction is an important stage for understanding information in a data set. Typical dimension reduction techniques, such as principal component analysis (PCA) [24], describe all the numerical variables contained within a data set in terms of a number of linear combinations (fewer than the original number of features) of these features. Although a widely used and appreciated method for reducing the number of dimensions within a data set, PCA is only valid for numerical features. In addition, a more transparent feature selection method is often required to remove redundant features of various types to reduce the size of the data set without losing potentially valuable information. Although a range of feature selection techniques exist because of the nature of the features in the TUDA data set and the prior knowledge that a large number of variables were likely to be highly correlated, a correlation analysis and clustering were used in this study to allow highly correlated features to be determined and redundant features to be removed. These methods also helped us to discuss, evaluate, and agree on the features to be retained in collaboration with the data gatekeepers and expert clinicians who had in-depth knowledge of the data. Further feature selection was not carried out as we elected to retain as many features as possible for use in training the classifiers. This section describes the feature selection techniques performed, and the results of feature selection are described in the Results section.

#### Manual Feature Selection

Manual feature selection was performed to remove features containing large amounts of missing data and, therefore, considered not useful for the analysis. Free-text variables that could not be encoded were also removed. On the basis of expert clinical knowledge, features deemed irrelevant to the study were removed, as well as a number of subjective features where a comparable, objective laboratory-obtained feature existed in the data set.

#### Correlation and Association

A correlation analysis is necessary before the development of classification models for 2 primary reasons: “Algorithms might ‘overfit’ predictions to spurious correlations in the data; multicollinear, correlated predictors could produce unstable estimates” [25] and “Perfectly correlated variables are truly redundant in the sense that no additional information is gained by adding them” [26]. In other words, as many machine learning algorithms rely on linearly independent variables, strongly correlated variables must be evaluated and removed to avoid unreliable results. Moreover, 2 variables that follow the same behavior add little to the information gained by the data set and thus are considered redundant. The correlation analysis allows the determination of highly correlated variables, which may undermine the consequential data analysis results. Owing to the difference in categorization of the variables within the data set, correlation coefficients were calculated for numerical-numerical pairs, whereas the strength of association was necessary for categorical-categorical variables and categorical-numerical variables. Correlations between numerical variables were calculated using the Spearman nonparametric correlation coefficient [27], the strength of association between categorical variables was calculated using the Cramér V statistic [28], and the coefficient of determination (R2) was calculated between categorical and numerical variables [29].

#### Clustering

Clustering is useful in feature selection [26] to analyze the data to find structural patterns. Clustering can be used together with correlation analysis to identify those variables that behave in a similar manner; thus, the information offered by the variables may prove redundant. Clustering of variables can take 1 of 2 forms: hierarchical, which outputs an informative hierarchy, and nonhierarchical, which divides the data into clusters, within which the variables may behave similarly. Owing to the nature of the information this study seeks to derive, the focus was placed on hierarchical clustering, illustrated specifically in the form of tree structures or dendrograms.

Ascendant hierarchical clustering can use a mixture of both numerical and categorical variables to arrange variables into homogenous clusters, that is, variables that are strongly related to each other [30]. The algorithm for finding these related clusters follows the concepts of PCA and multiple correspondence analysis (MCA). In PCA and MCA, the data set is analyzed to find new linearly independent variables to describe the same set of data. In this hierarchical clustering, these new synthetic variables are used as the center points of the clusters, and each original variable is then grouped according to its similarity to the cluster center, either using the sum of the correlation ratio, for numeric variables, or the squared correlation, for categorical variables.

### Machine Learning Techniques

**Figure 2:**
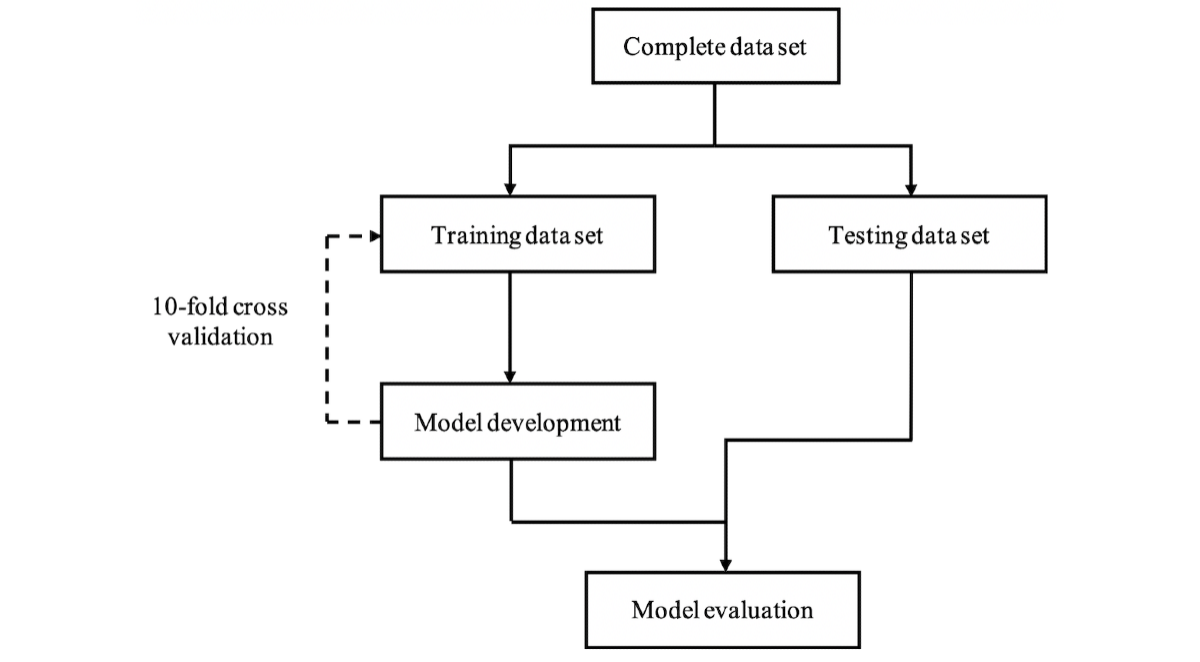
Model development and testing protocol.

Machine learning techniques are regularly employed for detecting patterns and dependencies within data, such as within health care data. Specifically, machine learning algorithms can be used to look for combinations of variables and generate rules within data that can be used to reliably predict outcomes [25]. This style of problem relies on classification algorithms, where predictor variables are used to predict an outcome or a class variable. These predictions are based on a training sample of the data, usually consisting of a random sample of about 70% to 80% of the available data. The developed model comprises rules based on these training data and then tested against the remaining data (Figure 2). The training procedure is repeated on a number of different subsets of the data to reduce the likelihood of overfitting the model. In this study, 10-fold cross-validation was used to measure the performance of classifiers. Initially, the data were split into a training set (75%) and an evaluation set (25%). The models were trained using the training set with 10-fold cross-validation applied (with a 90%/10% train/test split at each fold). The modeling techniques of decision trees, random forests, and Naive Bayes were selected for their ease of interpretability. It is crucial that the results of modeling in this study can be explained to clinical experts. The individual algorithms were developed using the R caret package, specifically using the train and predict functions. The evaluation data set was used to evaluate the performance of the model found to be optimal during training for each of the 3 respective techniques considered.

#### Decision Tree

Decision trees are one of the most common machine learning algorithms when using a combination of continuous and categorical variables, chosen for their computational efficiency and readability. The Classification and Regression Tree (CART) [31] algorithm, in particular, lends itself well to explanatory knowledge discovery [32] due to its transparency. CART decision trees are developed using a top-down recursive algorithm, where the data set is split into increasingly smaller subsets according to some predetermined metric, most commonly using either the Gini impurity index or a permutation importance measure. The measures used are described below. The rpart implementation of the CART decision tree algorithm in the R caret package was used in this study. This implementation automatically applies pruning, choosing a range of complexity parameters and automatically selecting the optimal model using the complexity parameter that provides the highest accuracy.

The resulting decision tree easily translates itself to a series of rules that can be used to classify the test data. The advantages of using a decision tree classifier lie in its ease of application, particularly as both numerical and categorical input variables require little to no preprocessing; its transparency for interpretation, as the resulting tree can be explained using Boolean logic; and its computational efficiency, particularly with large data sets. In addition, decision tree classification does not require domain knowledge or parameter setting [32]. However, traditional decision trees are also the least robust of the machine learning classification methods, as they are prone to overfitting and therefore rely substantially on the training data. Often, a small change in the training data can result in large changes in the developed tree. These shortcomings can be addressed using the random forest algorithm.

#### Random Forest

The random forest algorithm [33] works in a similar manner to decision trees, but where the CART algorithm results in a single tree, the random forest algorithm results in a *forest* of trees. Each of the maximal trees within the random forest will have been developed using a random subset of the predictor variables [34]. Each split within the tree is then calculated according to a given performance metric from only within this subset of variables. Typically, many trees are considered, thus reducing the prediction error, as the model prediction will reflect the average prediction across all trees. As a result, the random forest algorithm is considered robust, flexible, and highly suited to large data sets [35]. The random forest algorithm in the R caret package was used in this study. This implementation chooses a range of mtry parameters, where mtry is the number of variables available for splitting at each tree node, which have a strong influence on predictor variable importance estimates [36]. The mtry parameter providing the highest accuracy was used to select the optimal model.

#### Naïve Bayes

**Figure 3:**
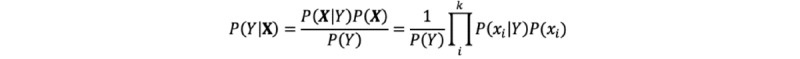
Naïve Bayes algorithm.

The Naïve Bayes algorithm for classification is based on Bayes’ theorem, which describes the most likely outcome (Y) based on k number of observations (X={x_1_,x_2_,…,x_k_}). This can be written as P(Y|X) and, as the algorithm is *naïve* and all variables are considered independent, is calculated using the equation in Figure 3.

The probability of an outcome P(Y); the probability of an observation being described by X, P(X); and the probability of an observation being described by X, given that they can be classed by Y, P(X|Y), can all be estimated using the given data set. For its use as a classifier, an observation is classified according to the most likely class based on the random variables the observation describes. A benefit of the Naïve Bayes classifier is its theoretical low error rate; however, based on the underlying independence of the variables, in practice, this may not be the case. The Naïve Bayes algorithm in the R caret package was used in this study.

### Importance and Accuracy Measures

#### Gini Impurity Index

**Figure 4:**
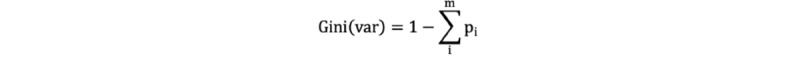
Gini impurity index.

The Gini impurity index describes the likelihood of an incorrect classification using a random variable (var) and is described mathematically as shown in Figure 4.

Here *p*_i_ is the probability of a correct classification according to m classes. By considering the variables resulting in a minimal Gini impurity index, this metric will therefore determine the best (most pure) variables to use to split the training data until a convergence criterion is met.

#### Permutation Importance

Permutation variable importance [33] is calculated by using the effect the variable has on the overall prediction performance. This performance can be predicted using the out-of-bag prediction error, calculated by taking the mean prediction error rate of those trees that did not include the specific variable [35].

#### Performance Evaluation

To compare the performance of each classification model, a variety of evaluation metrics were used. The accuracy, precision, recall, and *F*_1_ scores were computed. Precision, recall, and *F*_1_ scores take account of true and false positives and negatives, whereas accuracy considers only true-positives and true-negatives [37].

## Results

### Feature Selection

#### Manual Selection

Initially, 6 features deemed irrelevant for analysis were removed, including participant identification numbers and cohort category (which described the clinic from which the participants were selected). A total of 9 free-text variables and 9 variables with inconsistent questioning were removed. In addition, 94 subjective features were removed in favor of more objective laboratory-obtained results. Several of the removed subjective features had high numbers of missing values; therefore, removal of these in favor of subjective features assisted in handling missing data while ensuring that there was no information loss within the data set and data duplication was also minimized. For example, nutritional status based on blood analysis (eg, measurement of key vitamin biomarkers) was retained over self-reported dietary intake (eg, supplement and fortified food use).

#### Correlation and Association

**Figure 5:**
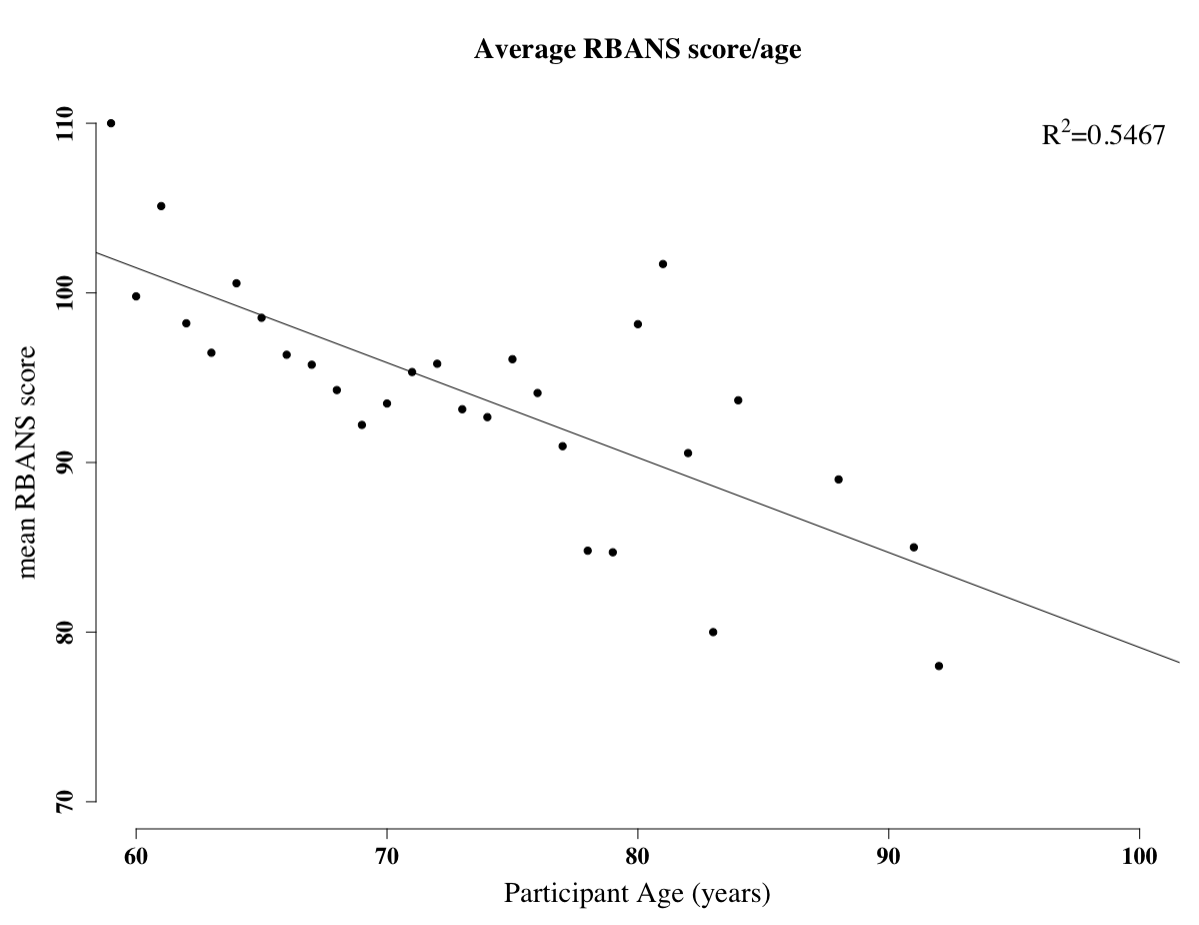
Mean Repeatable Battery for the Assessment of Neuropsychological Status (RBANS) score as a function of participant’s age. The graph shows a general decrease in the RBANS score as age increases. RBANS scores have been averaged by age; thus, each point represents the average score for any particular age. One outlier existed for age=86. This was removed and the R value recalculated accordingly.

Initial investigation into cognitive function with the TUDA data set, as measured using the RBANS score, highlights that as expected RBANS decreases with age (Figure 5).

Correlation and association analyses were carried out. The key results of this analysis are shown in (Multimedia Appendix 1). We observed a relationship between variables concerning follow-up questions within the questionnaire (eg, medication use and duration of use). On the basis of this, 41 features related to follow-up questions were removed. We also observed a high correlation between the use of specific medications (eg, bisphosphonate medications: Risedronate, Ibandronic acid, and Etidronate). These medications could be grouped into bone- and hormone-related categories, and therefore, we amalgamated each subset into a new variable. Specifically, 2 new variables were added for bone- and hormone-related medication, encompassing many types of bone medications, including bisphosphonates and hormone-related medications, from the original data set. This resulted in the removal of 30 features and the addition of 2 new features. Furthermore, scores for each assessment element of RBANS were removed and only the total score was retained. The total RBANS score was later used as the target variable in classification.

**Figure 6:**
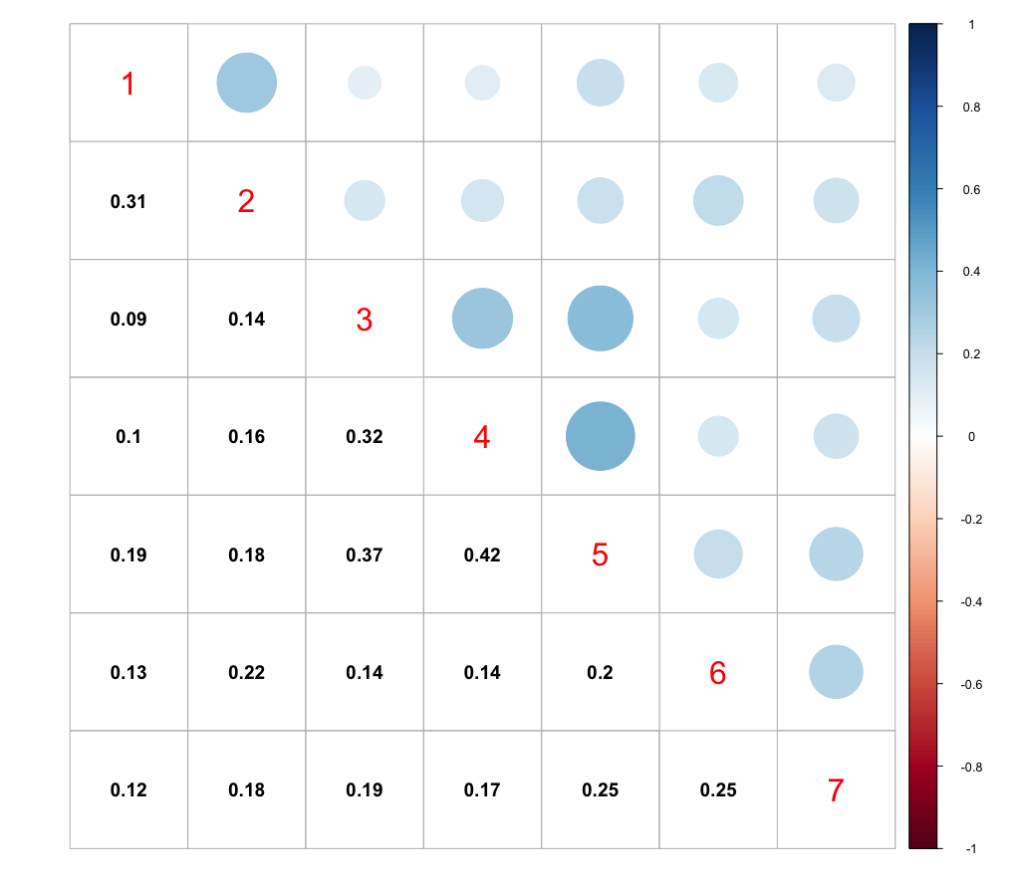
Correlation matrix using the Spearman (nonparametric) coefficient between participant test scores, ignoring observations with missing data. Variable descriptors are as follows: 1=Hospital Anxiety and Depression Scale total score; 2=depression questionnaire total score; 3=Mini-Mental State Examination total score; 4=Frontal Assessment Battery total score; 5=Repeatable Battery for the Assessment of Neuropsychological Status total score; 6=Physical Maintenance Scale total score; 7=instrumental activities of daily living total score.

We also removed the other neuropsychiatric test results (MMSE, FAB, Hospital Anxiety and Depression Scale, Centre for Epidemiological Studies Depression Scale) and functional test results (instrumental activities of daily living [IADL] and the physical self-maintenance scale [PSMS]) from the data set, as they are clinical assessment tools as opposed to individual predictor variables. This resulted in the removal of 72 additional features. The correlation matrix between these scores is shown in Figure 6.

The resulting subset of features following this stage of selection reduced the data set from 345 variables to 69 plus the class variable (RBANS score; Multimedia Appendix 2).

#### Clustering

**Figure 7:**
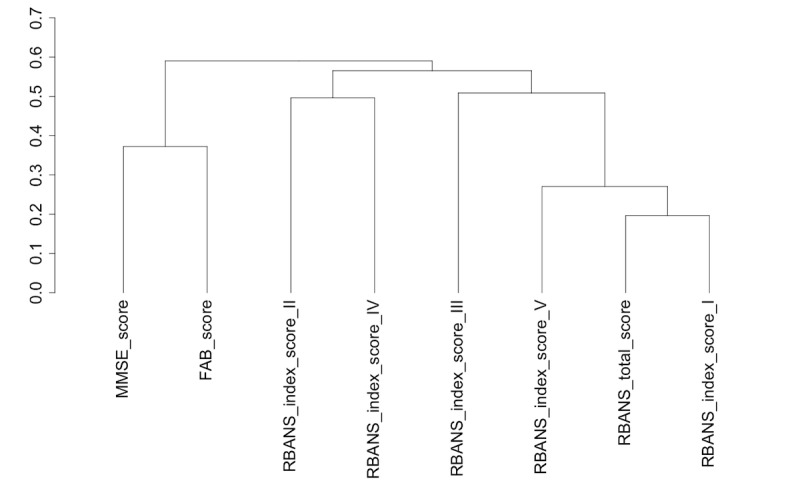
Hierarchical clustering of variables depicted as a dendrogram showing strong relationships between clinical assessment scores from the RBANS, FAB, and MMSE assessments. The variable descriptors are as follows: MMSE_score, Mini-Mental State Examination total score; FAB_score, Frontal Assessment Battery total score; RBANS_index_score_I, Repeatable Battery for the Assessment of Neuropsychological Status (RBANS) immediate memory score; RBANS_index_score_II, RBANS visuospatial constructional score; RBANS_index_score_III, RBANS language score; RBANS_index_score_IV, RBANS attention score; RBANS_index_score_V, RBANS delayed memory score; RBANS_total_score, RBANS total score.

**Figure 8:**
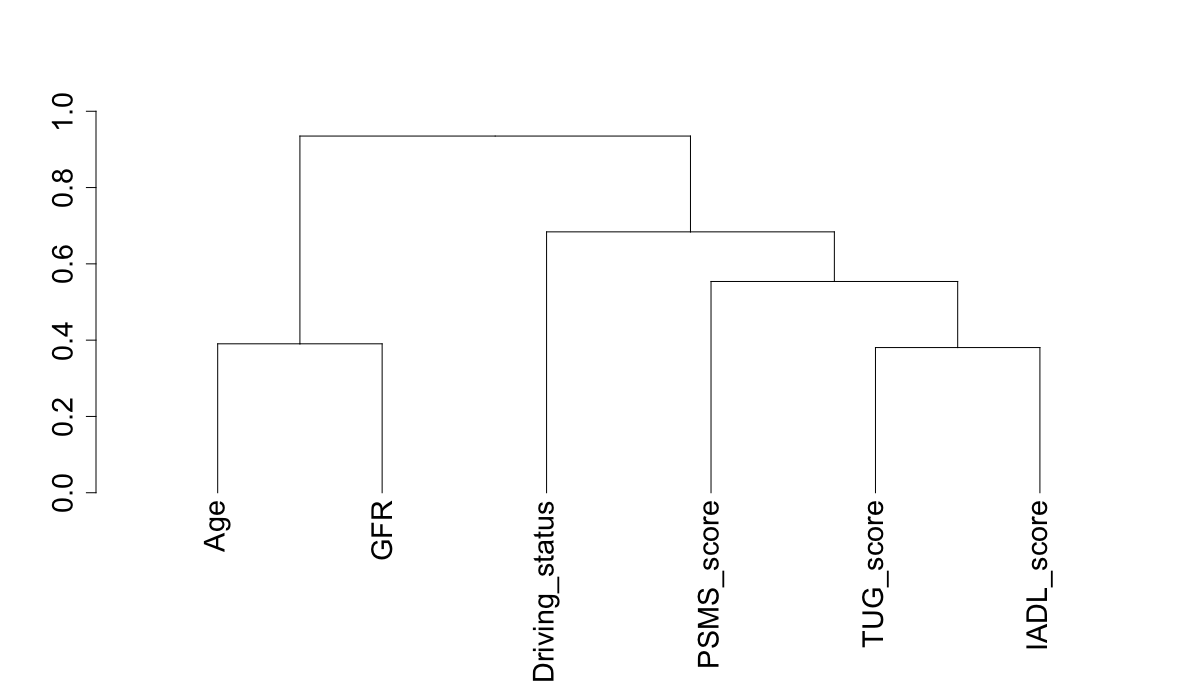
Hierarchical clustering of variables depicted as a dendrogram showing the close relation between a participant’s age and kidney function (glomerular filtration rate [GFR]), which together form a cluster with the physical diagnostic tests of IADL, TUG, and PSMS. The variable descriptors are as follows: age, participant’s age; GFR, kidney function; Driving_status, driving status; PSMS_score, Physical Maintenance Scale total score; TUG score, Timed Up and Go score; IADL_score, Instrumental Activities of Daily Living total score.

A cluster analysis was carried out using the ClustOfVar package within R Studio [30] to determine variable clusters and the strengths of their relationships. As expected, the scores from the clinical assessments, RBANS and its subcomponent tests, FAB and MMSE, are closely related (Figure 7). The participant’s age was closely related to kidney function, as indicated by the glomerular filtration rate (GFR), and together these form a variable cluster with the scores from the physical diagnostic tests of IADL, TUG, and PSMS indicating a relationship between these variables (Figure 8).

Following feature selection, the data set contained 69 features and 5186 observations; however, missing data still remained. To retain as much data as possible while minimizing the chance of statistical bias, participant records were imputed by replacing missing values with the average or expected value, in this case, according to the participant’s age and gender. As in other studies on the RBANS score [38], participants with visual (224 participants) or arthritic problems (1445 participants) were omitted as they would have been hindered from carrying out certain tasks within the test, and thus, their results may be unreliable, as were those displaying an MMSE score of <24 (647 participants). Upon removing the relevant records, 2869 observations remained.

### RBANS Classification

Classification models were utilized for 2 purposes: to discover if a model could be developed to predict a low RBANS score, representing poorer cognitive function, from the TUDA data set and to determine if the developed model could be used to identify key health, nutritional, and environmental predictors of these low scores.

The target variable in this analysis was the RBANS total score. For this analysis, the RBANS score was categorized using a data-driven clustering approach to find 2 natural groupings within the data identifying those with poorer cognitive performance as having an RBANS score <70 (assigned class *low*) and an RBANS score ≥70 was indicative of normal cognitive performance (assigned class *high*).

Class imbalance [39] within the data set was resolved using oversampling, in which a random sample of the smaller class was replicated until the class sizes were equal.

The supervised modeling techniques of decision trees, random forest, and Naïve Bayes were applied with 69 predictor variables (listed in Multimedia Appendix 2). The data set (n=2869) was split into a training set (2152/2869, 75%) and an evaluation set (717/2869, 25%). The models were trained using the training set with 10-fold cross-validation applied, and the results are shown in Table 2. For the decision tree model, the complexity parameter value of 0.020 for pruning was found to produce the highest accuracy. For the random forest model, the mtry value of 58 was found to produce the highest accuracy.

**Table 2:** Classification of the Repeatable Battery for the Assessment of Neuropsychological Status score performance measures when models were trained with 10-fold cross-validation (training set size=2152).

Classification technique	Accuracy, mean (SD)	Precision, mean (SD)	Recall, mean (SD)	*F*_1_, mean (SD)
Decision tree	0.737 (0.020)	0.795 (0.037)	0.643 (0.051)	0.709 (0.028)
Naïve Bayes	0.500 (0.000)	0.500 (0.000)	1.000 (0.000)	0.667 (0.000)
Random forest	0.990 (0.006)	1.000 (0.000)	0.981 (0.011)	0.990 (0.006)

The models were then evaluated using the held out 25% evaluation data set, and the accuracy of these models ranged from 60.4% using the decision tree to 87.7% using the random forest algorithm (Table 3). The random forest algorithm performed best in this comparison in terms of both accuracy and *F*_1_ score, with the decision tree algorithm performing the worst. This is expected in terms of robustness, specifically pertaining to problems with overfitting by the decision tree algorithm, which has been rectified somewhat using multiple trees within the random forest.

**Table 3:** Classification of the Repeatable Battery for the Assessment of Neuropsychological Status score performance measures when applied to the evaluation data set (training set size=2152; evaluation set size=717).

Classification technique	Overall accuracy	Precision	Recall	*F*_1_ score
Decision tree	0.604	0.926	0.596	0.725
Naïve Bayes	0.876	0.876	0.100	0.934
Random forest	0.877	0.882	0.992	0.934

**Figure 9:**
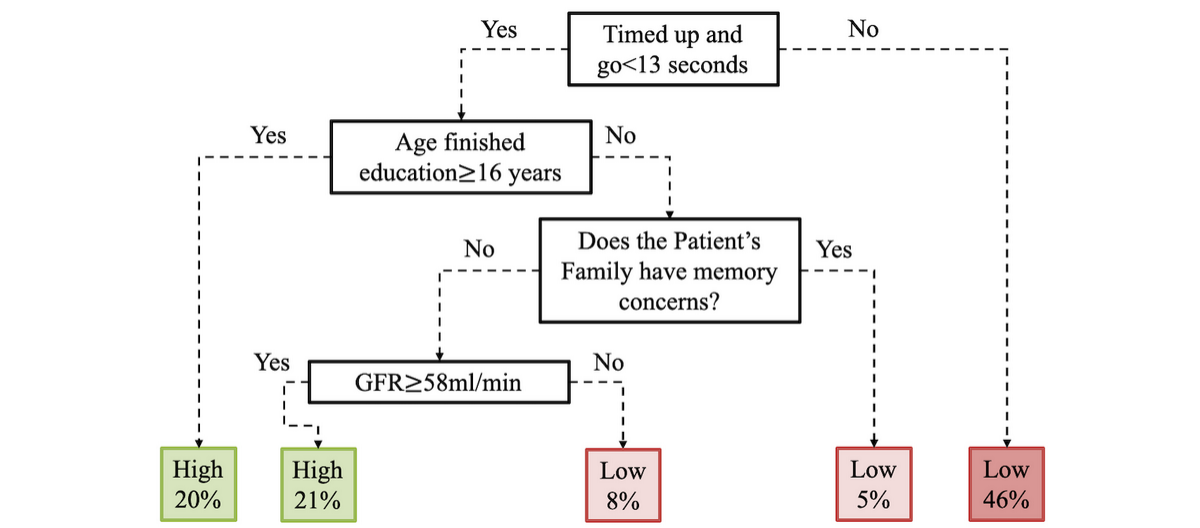
Decision tree classifier of the Repeatable Battery for the Assessment of Neuropsychological Status score. GFR: glomerular filtration rate.

The key predictors of the RBANS total score in the decision tree were as follows: participants’ scores from the TUG functional mobility test, representing the time a participant takes to get out of a chair, walk 3 m, turn around, and walk back to return to his or her original seated position; the age at which the participant stopped education; whether any family members were concerned about the participant’s memory; and the participant’s GFR, as shown in Figure 9. This decision tree predicted that a person who took under 13 seconds to perform the TUG test and stopped education after 16 years of age was classified as a *high* RBANS scorer (ie, indicative of normal cognitive performance). The decision tree classification model also highlights the importance of the TUG test alone; if a participant took longer than 13 seconds to perform the test, he or she was most likely to be a low scorer, indicative of poorer cognitive performance.

**Figure 10:**
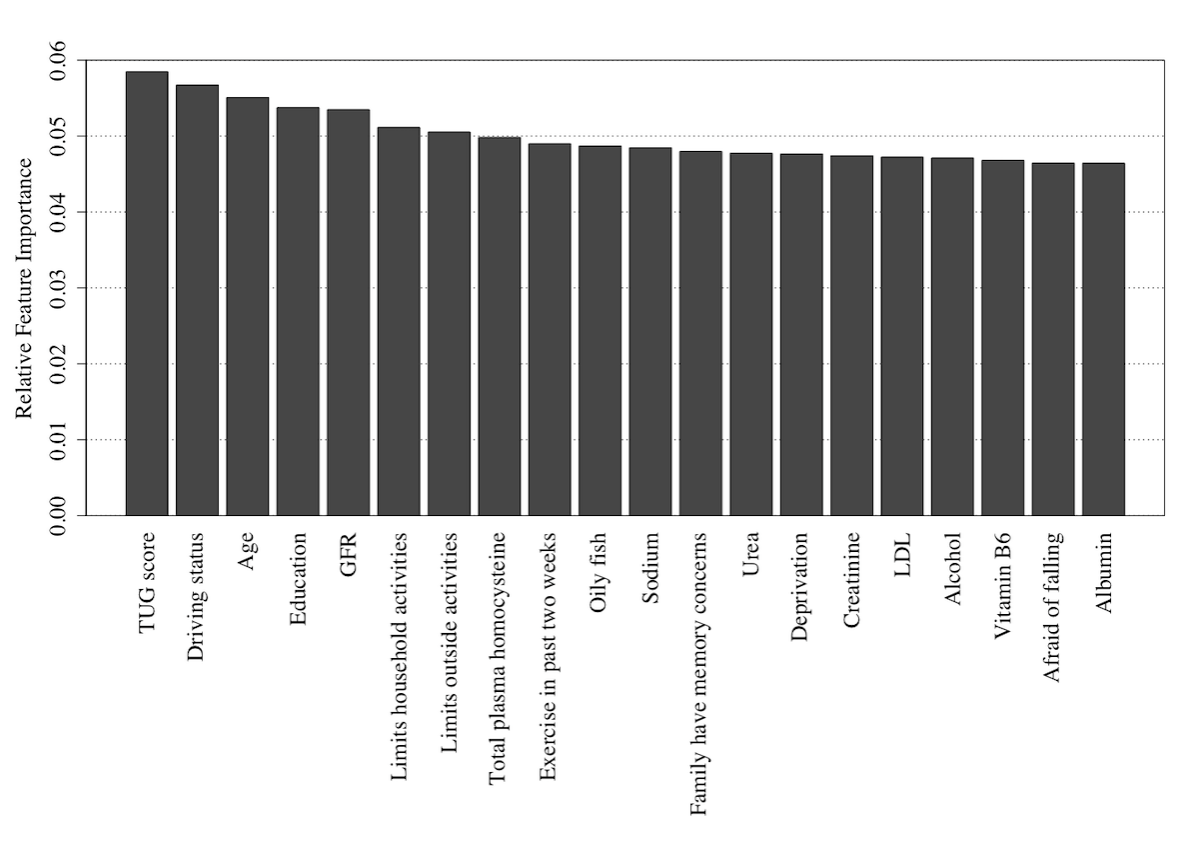
The 20 most important features for classification of the Repeatable Battery for the Assessment of Neuropsychological Status score as detected using feature permutation using a Naïve Bayes classifier. GFR: glomerular filtration rate; LDL: low-density lipoprotein; TUG: Timed Up and Go.

**Figure 11:**
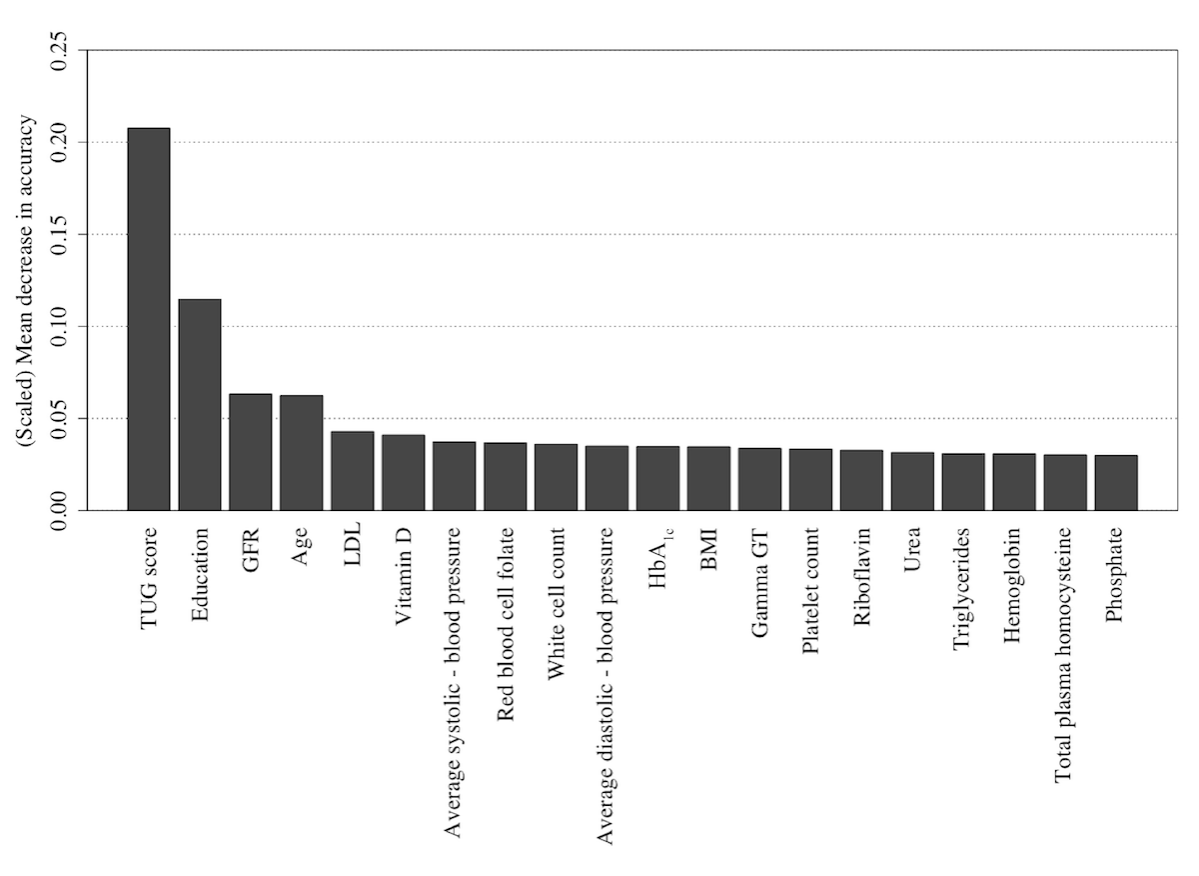
The 20 most important features for classification of the Repeatable Battery for the Assessment of Neuropsychological Status score as detected using feature permutation using a random forest classifier. GFR: glomerular filtration rate; HbA1c: glycated hemoglobin; LDL: low-density lipoprotein; TUG: Timed Up and Go.

Similarly, the Naïve Bayes and random forest algorithms also detect the TUG score, the age at which the participant stopped education, and the participant’s age as being highly informative features as shown in Figures 10 and 11 (see Multimedia Appendix 2 for feature descriptions) for Naïve Bayes and random forest models, respectively, with the Naïve Bayes algorithm adding a participant’s driving status and the random forest algorithm adding GFR to form the top 4 informative variables within these respective algorithms.

The informative nature of the 4 most important features determined by the most accurate classifier (random forest), as shown in Figure 11, was confirmed when these algorithms were rerun using only this subset of 4 features. In addition, 10-fold cross-validation was applied to train the model on the training data set (n=2152), with the results shown in Table 4. For the decision tree model, the complexity parameter value of 0.010 for pruning was found to produce the highest accuracy. For the random forest model, the mtry value of 2 was found to produce the highest accuracy. The models were then evaluated using the held out 25% evaluation data set. Training on the 4 most important features as determined by the random forest model resulted in a decrease in accuracy for the random forest model from 87.7% to 80.1% (Table 5). A larger reduction in accuracy was observed for the Naïve Bayes model, decreasing from 87.6% to 69.3%, whereas the decision tree model increased in accuracy from 60.4% to 72.5% when trained on this reduced data set compared with training on the original data set containing 69 variables.

**Table 4:** Classification of the Repeatable Battery for the Assessment of Neuropsychological Status score performance measures when models trained with 10-fold cross-validation (training set size=2152) and the 4 key variables: (1) age at which the participant stopped education, (2) the Timed Up and Go score, (3) the glomerular filtration rate measure, and (4) the participant’s age.

Classification technique	Accuracy, mean (SD)	Precision, mean (SD)	Recall, mean (SD)	*F*_1_, mean (SD)
Decision tree	0.688 (0.020)	0.702 (0.026)	0.655 (0.045)	0.677 (0.020)
Naïve Bayes	0.693 (0.012)	0.775 (0.021)	0.545 (0.026)	0.640 (0.018)
Random forest	0.929 (0.013)	1.000 (0.000)	0.857 (0.026)	0.923 (0.015)

**Table 5:** Classification of the Repeatable Battery for the Assessment of Neuropsychological Status score performance measures when models trained using the 4 key variables: (1) age at which the participant stopped education, (2) the Timed Up and Go score, (3) the glomerular filtration rate measure, and (4) the participant’s age when applied to the evaluation data set (training set size=2152; evaluation set size=717).

Classification technique	Overall accuracy	Precision	Recall	*F*_1_ score
Decision tree	0.725	0.928	0.732	0.819
Naïve Bayes	0.598	0.946	0.557	0.701
Random forest	0.801	0.878	0.889	0.883

### Classifying Cognitive Decline Using the Rate of Change in the RBANS Score

A subset (n=987) of TUDA study participants was reassessed using an identical protocol 5 to 7 years after the initial assessment. The result of this follow-up assessment enabled the creation of a new variable to add to the original TUDA data set for these 987 participants; the rate of change of the RBANS score (calculated using the equation in Figure 1). This variable would act as a measure of predicted cognitive decline (or improvement) over the 5- to 7-year follow-up period. The same classification models of decision tree, Naïve Bayes, and random forest were applied to the TUDA data (n=987), using the new *rate of RBANS change* as the classification variable. If the rate of change of a participant’s RBANS score was calculated as more than one half standard deviation below the mean rate of change of the RBANS score across the sample of participants, the participant was considered to have shown *acute decline* over time, otherwise the change in RBANS was considered *normal* or expected. The variable was normalized to adjust for differing periods of time between the first and second RBANS assessments (between 5 and 7 years) among participants. The data set (n=987) was split into a training set (740/987, 75%) and an evaluation set (247/987, 25%). The models were trained using the training set with 10-fold cross-validation applied, and the results are shown in Table 6. For the decision tree model, the complexity parameter value of 0.035 for pruning was found to produce the highest accuracy. For the random forest model, the mtry value of 2 was found to produce the highest accuracy.

**Table 6:** Classification of the Repeatable Battery for the Assessment of Neuropsychological Status score performance measures when models trained with 10-fold cross-validation (training set size=740).

Classification technique	Accuracy, mean (SD)	Precision, mean (SD)	Recall, mean (SD)	*F*_1_, mean (SD)
Decision tree	0.603 (0.045)	0.613 (0.053)	0.571 (0.151)	0.582 (0.083)
Naïve Bayes	0.499 (0.008)	0.499 (0.008)	0.997 (0.009)	0.665 (0.007)
Random forest	0.962 (0.026)	0.978 (0.035)	0.946 (0.031)	0.962 (0.028)

**Figure 12:**
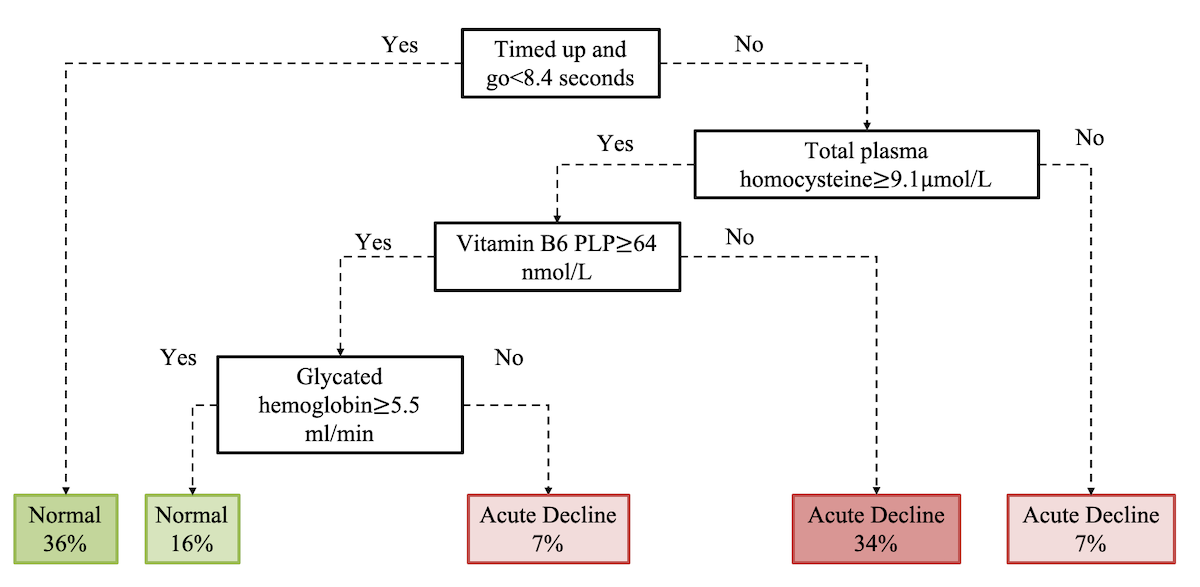
Decision tree classifier of rate of change of the Repeatable Battery for the Assessment of Neuropsychological Status score. PLP: vitamin B6 marker pyridoxal-5-phosphate.

The models were then evaluated using the held out 25% evaluation data set, and the results are shown in Table 7. Although the accuracy of these classification models is lower than that reported for the classification of the RBANS score, approximately 70% versus 90% for random forest classifiers, it nevertheless indicates the possibility of using our existing variables for predicting a perhaps pathological rate of cognitive decline to a reasonable level of accuracy. The decision tree performed the poorest; however, the information it provides (Figure 12) indicates that the TUG test score is again the most informative attribute, followed by the participant’s blood measures of total plasma homocysteine, vitamin B6 biomarker pyridoxal-5-phosphate (PLP), and glycated hemoglobin.

**Table 7:** Classification performance for rate of change of the Repeatable Battery for the Assessment of Neuropsychological Status score when applied to the evaluation data set (training set size=740; evaluation set size=287).

Classification technique	Overall accuracy	Precision	Recall	*F*_1_ score
Decision tree	0.547	0.735	0.605	0.664
Naïve Bayes	0.739	0.739	1.000	0.850
Random forest	0.702	0.735	0.933	0.822

**Figure 13:**
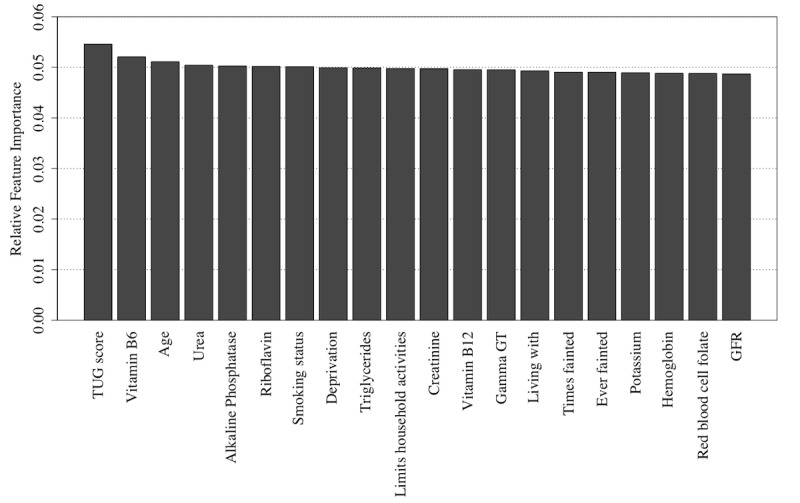
The 20 most important features for predicting rate of the Repeatable Battery for the Assessment of Neuropsychological Status change as detected using feature permutation using a Naïve Bayes classifier. Gamma GT: Gamma-glutamyl transferase; GFR: glomerular filtration rate; TUG: Timed Up and Go.

**Figure 14:**
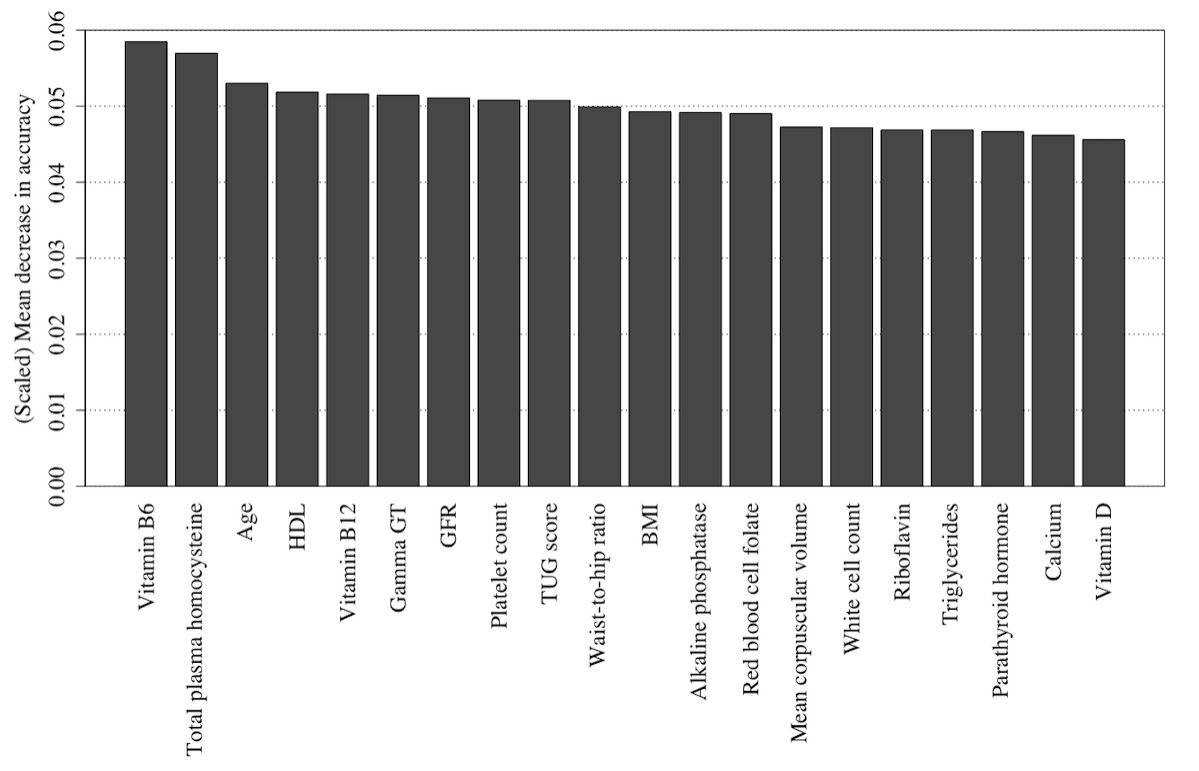
The 20 most important features for predicting rate of the Repeatable Battery for the Assessment of Neuropsychological Status change as detected using feature permutation using a random forest classifier. Gamma GT: Gamma-glutamyl transferase; GFR: glomerular filtration rate; HDL: high-density lipoprotein; TUG: Timed Up and Go.

Furthermore, using permutation importance measures (Figures 13 and 14, see Multimedia Appendix 2 for feature descriptions), it has been indicated that the same key variables for the classification of RBANS scores are no longer of such importance for the classification of rate of RBANS score change. Instead, the blood measures of PLP (vitamin B6 biomarker) and urea, coupled with the results of the TUG test and the participant’s age, are likely key predictors, particularly using the (best performing) Naïve Bayes algorithm (Figure 13).

## Discussion

### Principal Findings

The results of this study indicate that modeling of a variety of clinical, lifestyle, and sociodemographic factors using machine learning techniques may help predict poorer cognitive function in older people with a high level of accuracy (approximately 90%) and using a small number of noninvasive indicators. The approach is also useful, although slightly less accurate (approximately 70%), in predicting the rate of cognitive decline over a 5- to 7-year period with a small number of measures being the most influential health, nutritional, and environmental predictors. The results are important for clinicians and health service providers, especially at the early stages of engagement and diagnosis of cognitive dysfunction in older patients, by identifying those patients most in need of more intensive investigation. Furthermore, these findings may be useful for informing nutritional and lifestyle interventions aimed at maintaining brain health in the adult population.

The results presented here suggest that it may be possible for a health care professional to make an initial prediction (with a high level of confidence) of cognitive dysfunction using only a few short, noninvasive questions. Although the approach is not a diagnostic instrument for detecting the presence or absence of dementia, it has particular merit in that it could provide a very quick, efficient, and noninvasive screening method to help clinicians decide, at an early consultation stage, whether or not a patient should be investigated further using more in-depth cognitive assessment tools. Similarly, a recent study [14] used a machine learning approach to develop a gradient boosting machine classifier with the KLoSA data set [15], also identified sociodemographic, functional, and health-related factors, among others, as the most important predictors of cognitive impairment. The authors concluded that the model could be used to screen for cognitive impairment in a community health care setting. Using such an approach may offer potential benefits to both health service providers and older patients. It may provide time and cost savings for health service providers reducing the need for cognitive tests that are often laborious to administer (eg, it takes approximately 30 min to complete the RBANS assessment used in this study), and could potentially avoid testing of low-risk patients. As a result, any unnecessary stress associated with cognitive testing may be reduced or avoided in older adults. This study’s results also suggest that some additional invasive clinical measures may be required to identify those individuals at greatest risk of future cognitive decline, providing valuable information that could help clinicians design the most appropriate intervention and treatment strategies for patients on a case-by-case basis.

In the prediction of poorer cognitive performance, it is interesting to note that, in addition to participants’ age, the models identified noninvasive physical, behavioral, and socioeconomic variables over invasive clinical measures as the most influential predictors (with the exception of GFR), whereas the opposite was true for predicting the rate of change (with TUG being the exception). This suggests that nonclinical factors are much better in predicting poorer cognitive performance in older people, while clinical measures are needed to predict cognitive decline.

Machine learning methods produce the best classification models and predictive outcomes based on the quality and quantity (comprehensiveness) of the input variables. The potential for bias still remains, for example, when a key variable is missing from the data. Consequently, the results from the models need to be evaluated for theoretical and, in health outcome studies, clinical plausibility to determine their value and potential for real-world application [40].

In this study, all 3 models identified TUG and the age at which a participant stopped education as the most important predictive variables. In terms of plausibility, this is encouraging, as both these factors have been frequently identified and cited in the literature in large cohort studies as being important risk factors of cognitive dysfunction [6,41]. In support of these findings, we previously reported using a geodemographic analysis of this cohort that socioeconomic status, namely, area-based deprivation, was an important determinant of cognitive dysfunction alongside age, years of education, depression, and TUG test [42]. The emergence of the age a participant stopped education as the dominant variable from the socioeconomic cluster is particularly interesting as it has consistently been found to be the most important individual socioeconomic factor related to cognitive function across the life cycle [43]. Furthermore, 2 recent population-based longitudinal studies in the United States and the United Kingdom have indicated that higher educational attainment, particularly in early life, could help protect against a decline in cognitive function as people age [44,45]. Reduced physical function, measured using tools such as TUG, has also been associated with lower socioeconomic status [46] and cognitive dysfunction [47]. The TUG test reflects an individual’s strength and mobility, inherently assessing gait, balance, and, to a lesser degree, cognition and vision. It is a screening tool routinely used to assist clinicians in identifying patients at risk of falling [48]. A cutoff of ≥12 seconds is commonly applied to identify individuals at high risk of falls, but these cutoff levels are applied differently across various studies [49]. Within this study, a TUG score of >13 seconds was associated with poor cognitive performance, and a score of >8 seconds predicted future risk of cognitive decline. These selected predictors, and their associated split points, from the machine learning analytics, are consistent with other studies, where poor functional performance was correlated with lower executive function in patients with MCI and Alzheimer disease [50,51], and is associated with future dementia occurrence [52]. Moreover, the TUG test can be considered, in a sense, a global measure of body function. Poor performance has been associated with increased cardiovascular disease and mortality as well as all-cause mortality in older adults [53-55] and in patients with chronic kidney disease [56]. Additional predictors beyond the TUG score selected in the decision trees as informative are also linked with poor cognitive performance, including a measure of kidney function, GFR. Low GFR is associated with poorer cognitive performance [57], with a recent study reporting that individuals with impaired kidney function had lower cognitive performance compared with individuals with normal kidney function. Furthermore, in frail older adults with poor TUG scores, the severity of renal dysfunction is independently correlated with cognitive impairment [58]. Consequently, it is clear that the various machine learning approaches investigated in this study are identifying appropriate factors with known links to cognitive performance.

When the machine learning approaches were applied to identify the predictors of the rate of cognitive decline in TUDA participants over a 5- to 7-year follow-up period, vitamin B6 status (as measured by blood concentrations of the active form of the vitamin, PLP) at baseline emerged, after the TUG test, as one of the key predictors. High proportions of older adults in population-based surveys from the United States and Europe, including the United Kingdom, are reported to have deficient or low B6 status [59]. Vitamin B6 has a number of important biological roles, including immunomodulating effects. In clinical and population-based studies, blood B6 concentrations are found to be inversely associated with inflammatory conditions, neurodegenerative diseases, and depression and to predict the risk of cardiovascular disease and certain cancers [60]. Of note, vitamin B6 and related B vitamins (namely, folate, vitamin B12, and riboflavin) are required as cofactors in one-carbon metabolism, a series of essential reactions involving the transfer of one-carbon units for DNA synthesis and repair and homocysteine metabolism and in the methylation of phospholipids, proteins, DNA, and neurotransmitters [61]. There is a growing body of evidence indicating that one-carbon metabolism and related B vitamins may be important for maintaining cognitive health during aging. The majority of research to date has focused on folate and vitamin B12. Although vitamin B6 has been less extensively investigated, the findings of this study are in agreement with other observational studies. A low vitamin B6 status has been associated with cognitive dysfunction [62,63] and cognitive decline [64,65] in older people. A low vitamin B6 status was associated with cognitive decline in the Veterans Affairs Normative Aging Study [65]. More recently, a low baseline status of vitamin B6 was also associated with a greater-than-expected rate of cognitive decline in a cohort of community-dwelling older adults in Northern Ireland [64]. Of greater importance, a number of randomized controlled trials demonstrated that vitamin B6 supplementation in combination with other B vitamins reduces the rate of cognitive decline in older people [66,67] and a reduced rate of brain atrophy as measured using MRI [68]. Furthermore, other evidence from the TUDA study indicates that vitamin B6, along with folate and riboflavin, is associated with an increased risk of depression [7]. This machine learning approach has identified vitamin B6 as an important determinant of cognitive health in the TUDA study and, whilst biologically plausible and supported by other scientific evidence, the possible beneficial effects of vitamin B6 on cognitive health would need to be confirmed in randomized controlled trials.

What is very interesting from a clinical setting are the changes in the selected predictors within machine learning models when comparing the RBANS total score model versus the rate of change of the RBANS score model. The age at which a participant stopped education is a dominant predictor from the socioeconomic cluster in the RBANS total score model; however, it becomes an uninformative predictor of the rate of change of the RBANS score model and actually disappears from the models. This implies that while this socioeconomic factor is an important predictor of cognitive dysfunction (diagnosis), it is not important when predicting the rate of cognitive decline. Thus, while patients may start off on a different baseline due to socioeconomic predictors, their rate of cognitive decline is not influenced by these socioeconomic predictors.

Although this paper focuses on key health, nutritional, and environmental predictors of cognitive dysfunction and rate of change of cognitive function using machine learning techniques, as part of the project, the research team also sought input from personal and public involvement (PPI): patients, carers, and clinicians. This engagement focused on causation of cognitive dysfunction, particularly in relation to age, activity, and genetics, considered as measures of risk. This aspect of the work in terms of engagement with PPI, their expectations, and how these align with the findings of this work will be the focus of future research publications.

### Limitations

This study had several strengths and limitations. The main limitation is that the TUDA study is observational in design and thus residual confounding and reverse causality cannot be ruled out in this analysis. In addition, owing to the low instances of participants with poorer cognitive performance as indicated by an RBANS score below 70 (target class=*low*), this class was underrepresented within the training data set, and therefore, oversampling had to be performed to allow for more balanced classifier training. This artificial approach of boosting the number of samples was necessary for the classifier, but, coupled with the imputation of missing data, no new information would have been attained. This led to an imbalance between the precision and recall accuracy metrics, although this was remedied with the use of the *F*_1_ score. Generally, the algorithms performed well in the classification of the RBANS score. The decision trees performed the poorest, but as explained in the *Results* section, they were still capable of drawing out key and transparent information. Although an extensive comparison of classification approaches was not the focus of this study, we recognize that alternative variations of the algorithms used in this study exist, for example, C4.5 and C5.0 for decision trees as well as other learning algorithms such as neural networks and boosting algorithms. These alternative approaches may yield better results, and we intend to investigate these in the future while ensuring that the interpretability of results remains to be a key objective. In addition, the performance of the classifiers could have been improved using a dimension reduction technique such as PCA; however, this would have impacted the interpretability of the classifier, as was the objective of the study.

The main strength of this study is the utilization of data from the TUDA study, a large and comprehensively characterized cohort of community-dwelling older adults. Furthermore, a subset of the TUDA study cohort was reexamined 5 to 7 years later using standardized protocols at both time points. This enabled changes in cognition to be tracked over time and the rate of cognitive decline to be calculated compared with most observational studies that measure cognition at one time point only. The primary outcome of this study was based on the RBANS test, a sensitive neuropsychiatric battery for global cognitive assessment. As comprehensive data were available, this permitted objective laboratory measures over subjective measures of nutritional status to be included in the analytical models, thus providing more robust data on predictors of cognitive function.

### Conclusions

In conclusion, the derived classification models were able to identify a small number of key noninvasive predictors that are able to predict cognitive dysfunction and the rate of change of cognitive function with a high level of accuracy in the TUDA study. The TUG score, the age at which the participant stopped education, and whether or not the participant’s family reported memory concerns emerged as key predictors that could potentially be incorporated into a screening tool for cognitive dysfunction for health care professionals to identify individuals in need of further in-depth cognitive evaluation. Given the burden on health care resources, this could result in improvements in the efficiency of dementia screening and present cost and time savings for the relevant health professions. Furthermore, the results provide evidence to identify key targets that could be included in public health strategies aimed at prevention of dementia. Further investigation is necessary to test the accuracy of the identified predictors in other large cohorts and using other cognitive assessment tools. The TUDA data enable extensive opportunities for future investigations of the aging population.

## Appendix

Multimedia Appendix 1 Correlation and association matrix of Trinity-Ulster and Department of Agriculture dataset variables.

Multimedia Appendix 2 List of remaining quantitative and qualitative variables from the Trinity-Ulster and Department of Agriculture dataset and their descriptions following feature selection stages as determined by manual selection, correlation analysis and clustering.

## Abbreviations

ADNI: Alzheimer’s Disease Neuroimaging Initiative
AIBL: Australian Imaging Biomarkers and Lifestyle Flagship Study of Aging
CART: classification and regression tree
CRISP-DM: cross-industry process for data mining
FAB: frontal assessment battery
GFR: glomerular filtration rate
IADL: instrumental activities of daily living
KLoSA: Korean Longitudinal Study of Aging
MCA: multiple correspondence analysis
MCI: mild cognitive impairment
MMSE: Mini-Mental State Examination
MRI: magnetic resonance imaging
PCA: principal component analysis
PLP: vitamin B6 marker pyridoxal-5-phosphate
PSMS: physical self-maintenance scale
RBANS: Repeatable Battery for the Assessment of Neuropsychological Status
TUDA: Trinity-Ulster and Department of Agriculture
TUG: Timed Up and Go
