# Supplementary material for: Identifying Key Predictors of Cognitive Dysfunction in Older People Using Supervised Machine Learning Techniques: Observational Study
Source: JMIR Med Inform. 2020 Sep 16;8(9):e20995. doi: 10.2196/20995 (PMC7527918; doi:10.2196/20995)
Supplement: Multimedia Appendix 1 [file medinform_v8i9e20995_app1.doc]

**Multimedia Appendix 1**

**Definition of each numbered variable in correlation matrix: Correlation and association matrix of TUDA dataset variables:**

**
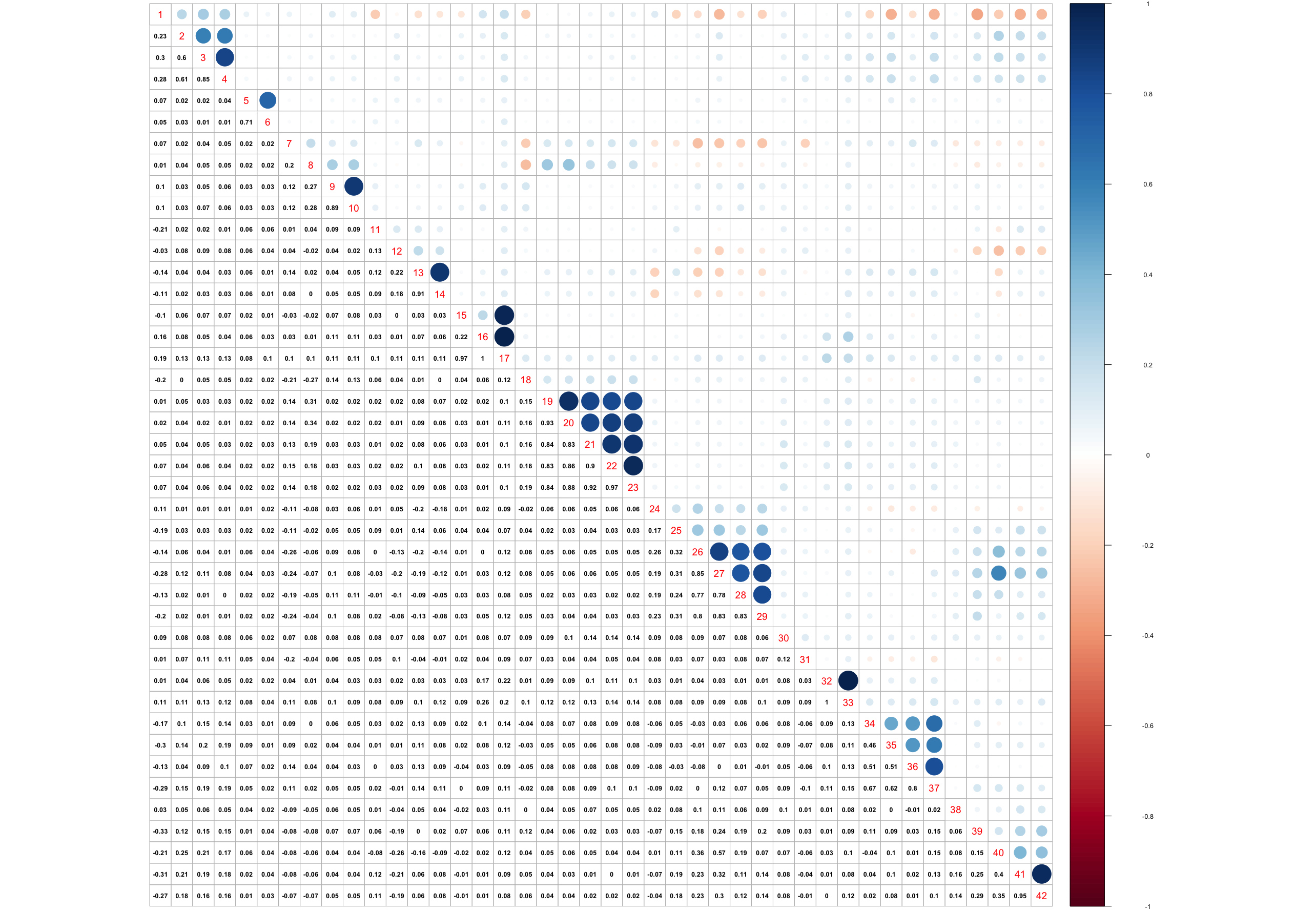
**

| **Variable Number** | **Variable Descriptor** |
| --- | --- |
|  |  |
| 1 | Age (Deciage) |
| 2 | Afraid of falling |
| 3 | Limits household activities due to fear of falling |
| 4 | Limits outside activities due to fear of falling |
| 5 | Family history of cancer |
| 6 | Family history of presenile dementia |
| 7 | Total Vitamin D |
| 8 | Red blood cell folate |
| 9 | Consumes folic acid fortified food |
| 10 | Consumes B12 fortified food |
| 11 | Calcium (Ca) |
| 12 | Platelet count (PLT) |
| 13 | Total cholesterol |
| 14 | Low-Density Lipoprotein (LDL) |
| 15 | Number of times participant felt like fainting in past year |
| 16 | Whether the participant has ever felt like fainting |
| 17 | Number of times participant has fainted |
| 18 | Riboflavin (B2) supplement user |
| 19 | B vitamin supplement user |
| 20 | Folic acid supplement user |
| 21 | B12 oral supplement user |
| 22 | B6 supplement user |
| 23 | Riboflavin (B2) supplement user |
| 24 | HbA1c |
| 25 | Triglycerides |
| 26 | Waist measurement (cm) |
| 27 | Weight (kg) |
| 28 | Hip measurement (cm) |
| 29 | Body mass index (BMI) |
| 30 | Country where participant’s father was born |
| 31 | Alkaline phosphatase |
| 32 | Whether participant has ever fainted |
| 33 | Number of times participant felt like fainting |
| 34 | RBANS language score |
| 35 | FAB score |
| 36 | RBANS immediate memory score |
| 37 | RBANS total score |
| 38 | Average systolic blood pressure |
| 39 | Sodium (Na) |
| 40 | Height (cm) |
| 41 | Haemoglobin (Hb) |
| 42 | Haematocrit (HCT) |
