# Supplementary material for: Identifying Key Predictors of Cognitive Dysfunction in Older People Using Supervised Machine Learning Techniques: Observational Study
Source: JMIR Med Inform. 2020 Sep 16;8(9):e20995. doi: 10.2196/20995 (PMC7527918; doi:10.2196/20995)
Supplement: Multimedia Appendix 2 [file medinform_v8i9e20995_app2.doc]

**Multimedia Appendix 1**

List of remaining quantitative and qualitative variables from the TUDA dataset and their descriptions following feature selection stages as determined by manual selection, correlation analysis and clustering

| **Variable** | **Description** | **Variable Type** |
| --- | --- | --- |
|  |  |  |
| Deciage | Age of participant | Numerical |
| WaistHip | Calculated Waist:Hip ratio | Numerical |
| Timedupngo | Timed Up and Go test results | Numerical |
| Bodymassindex | Calculated body mass index (BMI) | Numerical |
| AverageSystolic | Average systolic blood pressure reading | Numerical |
| AverageDiastolic | Average diastolic blood pressure reading | Numerical |
| Agefinishededucation | Age participant finished education | Numerical |
| Totalunitsperweek | Number of units of alcohol the participant consumes per week | Numerical |
| WCC | White cell count (Biochemistry; full blood profile) | Numerical |
| Hb | Haemoglobin (Biochemistry; full blood profile) | Numerical |
| MCV | Mean corpuscular volume (Biochemistry; full blood profile) | Numerical |
| PLTS | Platelet count (Biochemistry; full blood profile) | Numerical |
| Urea | Urea (Biochemistry; renal profile) | Numerical |
| Creatinine | Creatinine (Biochemistry; renal profile) | Numerical |
| Albumin | Albumin (Biochemistry; liver profile) | Numerical |
| GammaGT | Gamma GT (Biochemistry; liver profile) | Numerical |
| Na | Sodium (Biochemistry; electrolytes) | Numerical |
| K | Potassium (Biochemistry; electrolytes) | Numerical |
| Ca | Calcium (Biochemistry; electrolytes and bone profile) | Numerical |
| Po3 | Phosphate (Biochemistry; electrolytes) | Numerical |
| AlkPhos | Alkaline Phosphatase (Biochemistry; liver and bone profile) | Numerical |
| LDL | Low-Density Lipoprotein (Biochemistry; lipid profile) | Numerical |
| HDL | High-Density Lipoprotein (Biochemistry; lipid profile) | Numerical |
| Triglycerides | Triglycerides (Biochemistry; lipid profile) | Numerical |
| CRP | High sensitivity C-reactive protein (Biochemistry; inflammatory biomarker) | Numerical |
| HbA1C | Glycated haemoglobin (Hb Biochemistry; marker of diabetes) | Numerical |
| PTHpgml | Parathyroid Hormone (Biochemistry; bone profile) | Numerical |
| GFRmlmin | Glomerular filtration rate (Biochemistry; renal profile) | Numerical |
| Vitamin_D | Total vitamin D (D2 +D3) (25(OH)D) (Biochemistry) | Numerical |
| tHcyumolL | Total plasma homocysteine (Biochemistry; nutritional biomarker of B-vitamin status) | Numerical |
| TotalserumB12pmolL | Total vitamin B12 (Biochemistry; nutritional biomarker of vitamin B12 status) | Numerical |
| RCFnmolL | Red Cell Folate (Biochemistry; nutritional biomarker of folate status) | Numerical |
| VitaminB6markerPLPnmolL | Vitamin B6 PLP (Biochemistry) | Numerical |
| RiboflavinmarkerEGRac | Riboflavin marker (EGRac) (Biochemistry) | Numerical |
| Totalscale | RBANS score | Numerical |
| Gender | Gender of participant | Nominal |
| Living_with | Who the participant is currently living/sharing accommodation with | Nominal |
| Marital_status | Marital status | Nominal |
| Driving_status | Car driving status | Nominal |
| ABICarotidarterydopplers | Whether the participant ever had an ABI/carotid artery doppler performed | Nominal |
| Carotidendarterectomy | Whether the participant ever had a carotid endarterectomy performed | Nominal |
| Bypassoperations | Whether the participant ever had a Bypass performed | Nominal |
| GI_disease | Whether the participant ever had a gastrointestinal disease | Nominal |
| Familyhistoryofcancer | Whether the participant (to their knowledge) has  a family history of cancer | Nominal |
| Familyhistoryofstroke | Whether the participant (to their knowledge) has a family history of stroke | Nominal |
| Familyhistoryofheartdisease | Whether the participant (to their knowledge) has a family history of heart disease | Nominal |
| Familyhistoryofpreseniledementia | Whether the participant (to their knowledge) has a family history of presenile dementia | Nominal |
| Familyhistoryofseniledementia | Whether the participant (to their knowledge) has a family history of senile dementia | Nominal |
| Falleninlastyear | Whether the participant has (to their knowledge) fallen in the last year | Nominal |
| Caretakenwhenrising | Whether the participant has to be careful not to stand up too quickly when rising from a sitting or lying position | Nominal |
| Dizzywhenstandingtooquickly | Whether the participant feels dizzy if they stand up too quickly | Nominal |
| Everfainted | Whether the participant has ever fainted | Nominal |
| Feltlikefainting | Whether the participant ever felt like they were going to faint but did not | Nominal |
| Afraidoffalling | Whether the participant is afraid of falling | Nominal |
| Limithouseholdactivities | Whether the participant limits any household activities because they are afraid they might fall | Nominal |
| Limitoutsideactivities | Whether the participant limits any outside activities because they are afraid they might fall | Nominal |
| Exerciseinpasttwoweeks | Whether the participant has done any exercise in the past two weeks | Nominal |
| Oilyfish | Whether the participant consumes oily fish | Nominal |
| Smokingstatus | Smoking status of the participant | Nominal |
| Visualimpairment | Whether the participant has a visual impairment | Nominal |
| Memoryconcernsself | Whether the  participant has any concerns with regard to their memory | Nominal |
| Memoryconcernsfamily | Whether the participant’s family has any concerns with regard to their memory | Nominal |
| Parentshadhipfracture | Whether the participant’s parents ever had hip fracture | Nominal |
| SufferwithRheumatoidArthritis | Whether the participant suffers from rheumatoid arthritis | Nominal |
| SufferwithOsteoporosis | Whether the participant suffers from osteoporosis | Nominal |
| EversufferedfromEpilepsy | Whether the participant suffers from epilepsy | Nominal |
| ad_mn_groups | Deprivation quintile category of the census enumeration area where the participant lives, where 1 = least deprived to 5 = most deprived | Nominal |
| BoneMed | Whether the participant takes at least one of Protelos/Bisphosphonates/Alendronate/Risedronate/Ibandronic/Etidronate/Zoledronic | Nominal |
| HormMed | Whether the participant takes one of Aromatase/Arimidex/Femara/Zoladex/Prostap/Gonapeptyl | Nominal |
